# Implementation & yield of upfront genomic profiling in a clinical prostate cancer diagnostic pathway

**DOI:** 10.1111/bju.16101

**Published:** 2023-07-10

**Authors:** Charlie Massie, Vincent Gnanapragasam, Tristan Barrett, Anne Warren, Shubha Anand, Alexandra Keates, Simon Pacey

**Affiliations:** 1 Cambridge Prostate Cancer Collaborative, University of Cambridge, Cambridge United Kingdom; 2 Early Detection Programme, CRUK Cambridge Cancer Centre, Cambridge United Kingdom; 3 Department of Oncology, University of Cambridge, Cambridge United Kingdom; 4 Division of Urology, Department of Surgery, University of Cambridge, Cambridge United Kingdom; 5 Cambridge Urology Translational Research and Clinical Trials Office, Cambridge Biomedical Campus, Addenbrooke’s Hospital, Cambridge, UK; 6 Department of Radiology, University of Cambridge, Cambridge United Kingdom; 7 Department of Pathology, Addenbrookes Hospital, Cambridge, Cambridge United Kingdom; 8 Cancer Molecular Diagnostics Laboratory, CRUK Cambridge Cancer Centre, Cambridge United Kingdom

Recent landmark studies have shown a survival benefit from using genomic profiling to guide targeted personalised therapy in men with metastatic castrate refractory prostate cancer (mCRPC) (1). The point at which genomic profiles should be acquired in the prostate cancer pathway however remains unclear. Primary high-grade and/or locally advanced prostate cancer has a significant relapse rate despite radical therapy with 30-60% developing distant metastases, cancer specific and all-cause mortality within a median of 6.5 years follow-up (2). The UK National Prostate Cancer Audit has published that more than a half of all new cancers diagnosed present with such aggressive (NICE Cambridge Prognostic Group 4-5: CPG4-5) or de novo metastatic disease (3). This represents a large demographic of men with a high probability of disease progression to the mCRPC state and in whom knowledge of their genomic status may help in managing their treatment journey. Here we explored evaluating genomic mutation yield upfront at first diagnosis in these men by synergising data from pre-biopsy imaging, guided biopsies and modern prognostics.

Patients referred to a standard NHS diagnostic service between Jan 2019 -Dec 2021 were screened using the pre-biopsy MRI report and presenting PSA (4). Eligible men (REC ethics 03/018) included those with raised PSA and MRI PI-RADS 4-5 and/or evidence of ≥T3 disease or de novo metastatic disease. Biopsies were taken from image-targeted sites as well as systematic biopsies (no extra or specific research samples added) and prepared as standard (formalin fixed paraffin embedded – FFPE). A uro-pathologist marked all cancer areas from target and non-target samples. Surplus to diagnosis FFPE sections matching the marked areas were sent for sequencing. The methods for extraction, DNA isolation and sequencing are detailed in the supplementary material. For this study a 350 gene capture panel (TWIST Biosciences) was used.

62 men were recruited of whom 52 (84%) had cancer samples sufficient and met quality for sequencing. Cohort details are in supplementary table S1. 37/52 (71%) were either ≥Cambridge Prognostic Group (CPG) 4-5 (n=30) or had metastatic disease (n=7) at diagnosis. These data confirm high yields of aggressive cancers from pre-biopsy selection (PSA and MRI) and that samples for sequencing can be achieved without disrupting the standard diagnostic pathway. DNA Damage Repair (DDR) pathogenic mutations were detected in 5 individuals with 4 harbouring BRCA2 (1 germline) and 1 ATM (supplementary Table S2). All were men with CPG 4-5/ metastatic disease representing 14% (5/37) of this sub- cohort. Currently the 4 BRCA2 are potentially actionable mutation which Poly (ADP-ribose) polymerase (PARP) inhibitor (PARPi) could target (5). A further 4 cases were called BRCA2 or ATM loss by the algorithm but were of uncertain clinical significance. In the PI3K-Akt pathway, PTEN mutations were identified in 4 men and a further 3 were present in phosphatidylinositol 3-kinase (Pi3K) related gene subunits with 1 mutation in MTOR (total 8/52, 15%) (Table S2). Seven were in men with CPG 4-5 or metastatic disease (19%, 7/37 of this sub-cohort). Based on eligibility for use of the novel AKT inhibitor Capiversetib within the Capitello-281 trial (NCT04493853), mutations in PTEN and PiK3CA might be actionable, with an AKT inhibitor representing 5/52 (10%) of the whole cohort and 4/37 (11%) of the CPG4-5/metastatic sub-cohort. Other pathogenic mutations called are shown in Table 2 of which the commonest was p53 (6/52, 12%).

Focusing on the 37 men with CPG4-5/metastatic disease, all but two had combined androgen deprivation therapy (ADT) and radiotherapy or systemic therapy alone (ADT and chemotherapy). Follow up was available for a median of 26 months (range 15-35). In this CPG4-5/metastatic sub-group; overall there were 12/37 (32%) progression events; 9 new or worsening metastases and 4 who progressed and died of prostate cancer. Three others developed rapid biochemical relapse after first-line treatment. Of the 5 men with DDR pathway mutations, 2/5 progressed; one with a BRCA2 mutation died from disease and another developed new metastasis. Interestingly 1 of the men with ATM loss of uncertain significance, relapsed rapidly after surgery. Of the 6 men with any Pi3K-Akt mutation there was 1 death from prostate cancer and 1 progression despite treatment. Combining men with either a DDR or a Pi3K pathogenic pathway mutation 4/11 (36%) progressed and/or died of disease (1 man had both BRCA2 and PI3K mutation). In men without any reported DDR or PI3K mutations (pathogenic or uncertain), 5/22 (23%) progressed in the follow-up period.

It is now clear that specific genomic signatures add value in management decisions and impact disease trajectory and survival outcomes (6). Falling costs further support the notion that sequencing could be cost-effective within a publicly funded health care system. Recognising this, the UK NHS Genomics in Medicine initiative now offers selected DDR mutation profiling as part of commissioned cancer services (7). The challenge to widespread implementation though is the sheer number of men diagnosed annually, prior inability to accurately sample significant disease and uncertainty on which men benefit. The advent of MRI guided biopsies, advances in FFPE sequencing and a better understanding of disease prognosis has overcome many of these issues. We exploited these innovations in a 4-step process to optimise cohort selection (Figure 1):

(i) Targeting men with high PSA and/or MRI defined high probability lesions
(ii) Histology identification of tissue to be profiled at routine pathology assessment
(iii) Assign prognostic/metastatic category at MDT and determine likely outcomes vs competing morbidity for case selection
(iv) Sequencing of those men with a high likelihood of progression or cancer mortality –: CPG4-5/metastatic disease

We have shown that these steps are possible in a routine NHS diagnostic pathway and offers a rationale way to maximise the detection of potentially actionable mutations while avoiding over-investigation. Using this method DDR and PI3K mutations were detected in 11% and 19% in CPG4-5/metastatic disease and these men seemed to be over-represented in cases that progressed over the short term (observational data).

Our study is primarily descriptive as our aim was to assess feasibility and yield and we did not attempt to determine cost effectiveness. Nevertheless, as BRCA1/2 is already commissioned by NHS Genomics incorporating routine profiling is already possible under NHS provisions. Any tests however are only of benefit if it will alter management. In this regard use of PARPi drugs for treatment of BRCA mutant tumours (including metastatic prostate cancer) are now approved by NICE (as well as the European Medicines Agency and US FDA) (6). It could be argued that waiting for men to develop mCRPC before profiling may further reduce the numbers sequenced. However our approach abrogates issues including having to search off-site archives, repeat sectioning and reporting and degrading DNA quality from stored FFPE. Furthermore *a priori* knowledge of BRCA status may allow modifications in earlier stages of management and future proof for evolutions in genomic testing (including trials). BRCA2 status for instance is already an independent predictor of poor survival in the Predict Prostate algorithm (8).

In summary we report a pragmatic method to optimise selection of men for molecular profiling of prostate cancer at the point of diagnosis. Working within standard diagnostic pathways our method could be adopted by any NHS (or other) unit without much resourcing. The upfront information could be used not only for future drug selection, but also influence decision making and personalised follow-up.

## Supplementary Material

Supplementary File

## Figures and Tables

**Figure 1:**
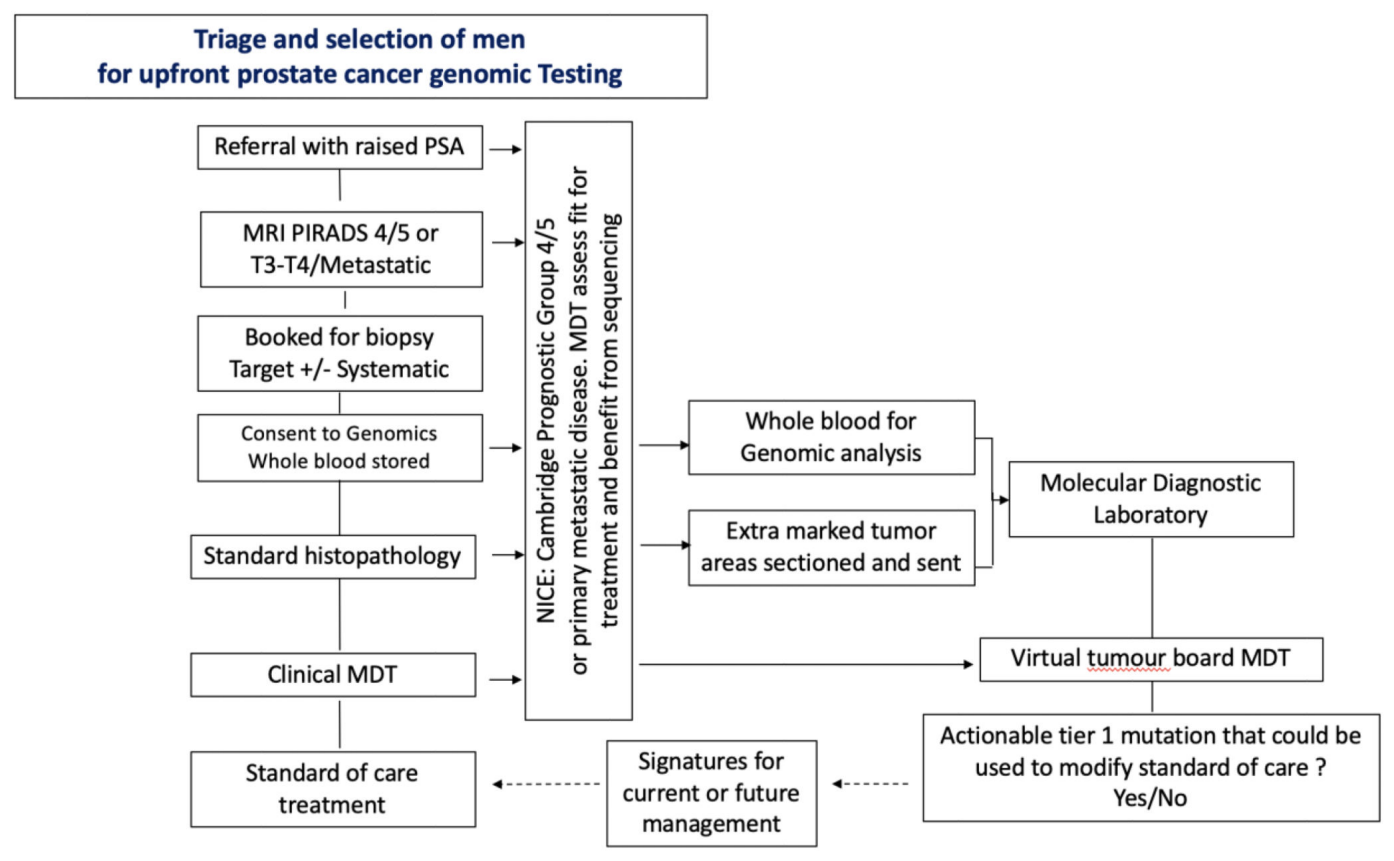
Patient flow and selection of cases for molecular profiling within a real-world standard of care prostate cancer diagnostic and management pathway
