# Supplementary material for: Implementation & yield of upfront genomic profiling in a clinical prostate cancer diagnostic pathway
Source: BJU Int. Author manuscript; Available in PMC 2023 Nov 1. (PMC7615268; doi:10.1111/bju.16101)
Supplement: Supplementary File [file EMS189929-supplement-Supplementary_File.docx]

**Supplementary methods**

**DNA isolation and Targeted Next Generation Sequencing**

Surplus to diagnosis FFPE sections matching the marked areas were sent for sequencing where material was acquired from the slides pooled for extraction to give a global estimate of cancer profile. DNA was extracted from buffy coats (for germline sample) or FFPE specimen (somatic sample) using the Qiagen DNA extraction kits (QIAsymphony DNA Mini Kit, QIAamp Blood DNA Mini Kit and QIAamp DNA FFPE Tissue kit). Libraries were prepared from 100ng of DNA using a custom capture based 350 gene panel (Total Size: 1,455,082 base pairs) from TWIST Biosciences and were sequenced on Illumina HiSeq4000 in an on-site GCLP accredited facility (Cambridge Molecular Diagnostics Laboratory). Sequencing data was analysed using an in-house bioinformatics pipeline (version 0.41) which utilizes the following main algorithms: bwa-mem (hg38 alt contig aware alignment), samtools (PCR Duplicates removal), GATK (Base Quality Score, Recalibration, InDel Realignment), GATK's Haplotype Caller (Germline Variant Calling), GATK's MuTect2 (Somatic Variant Calling), GATK (CNV Calling), Delly (Non-CNV SV Calling) and Annovar (Variant Annotation) following best practices. This panel focused mainly on exonic variants with little coverage of intronic regions. The Minimum coverage to call variants was 200x, and the targeted coverage was 500x. The sensitivity of this assay is 99% down to 3% of mutant alleles over a wild-type background for SNVs/INDELS. CNV analysis was not performed, and the algorithm may not have detected complex deletions, duplications, and large genomic rearrangements. For tissue sample FFPE analysis all common variants present in population databases (1,000 genome, exac and gnomAD) at variant allele frequency (vaf) >1% were removed as well as all known benign variants. In somatic analysis, germline mutations present in tumour were not filtered out and mutations which were germline are indicated in the Table 2.

|  | **Cohort n=52** |
| --- | --- |
| **Age (years)** |  |
| Mean | 68.2 |
| Median | 68.5 |
| Interquartile Range | 63-75 |
|  |  |
| **PSA (ng/ml)** |  |
| Mean | 19.2 |
| Median | 10.02 |
| Interquartile Range | 5.9-20.7 |
|  |  |
| **Grade Group** |  |
| GG1 | 7 |
| GG2 | 11 |
| GG3 | 8 |
| GG4 | 9 |
| GG5 | 17 |
|  |  |
| **MRI Stage** |  |
| T2 | 18 |
| T3a | 15 |
| T3b | 18 |
| T4 | 1 |
|  |  |
| **MRI Likert score** |  |
| 4 | 8 |
| 5 | 44 |
| **Cambridge Prognostic Group*** |  |
| CPG1 | 7 |
| CPG2  CPG3 | 6  2 |
| CPG4 | 8 |
| CPG5  **Metastatic at diagnosis** | 22  7 |
|  |  |
| **Metastasis sites at diagnosis (n=7)**  Bone metastasis  Lymph node metastasis | 4  7 |
| **First treatment received** |  |
| Primary androgen deprivation +/- chemotherapy/ | 6 |
| Radiotherapy + androgen deprivation therapy  Radical prostatectomy | 35  3 |
| Active Surveillance/Watchful waiting | 6 |
| Other | 2 |
|  |  |

**Supplementary Table 1** – Demographics of the study population. *NICE Cambridge Prognostic Group (<https://www.nice.org.uk/guidance/ng131/chapter/Recommendations#localised-and-locally-advanced-prostate-cancer>)

|  |  | | **Actionable*** | **% of cases (n=52)** | **% of** $\boldsymbol{\geq}$**CPG4 disease (n=37)** |
| --- | --- | --- | --- | --- | --- |
|  | |  |  |  |  |
| **DDR** | |  |  |  |  |
| BRCA2 | | 4 (1^#^) | Yes | 8% | 11% |
| *Other* | |  |  |  |  |
| *ATM* | | *1* | - |  |  |
|  | |  |  |  |  |
| **All DDR** | | 5 | - | 10% | 14% |
|  | |  |  |  |  |
|  | |  |  |  |  |
| **Pi3K-AKT** | |  |  |  |  |
| PTEN | | 4 | Yes | 8% | (n=3) 8% |
| *Other* | |  |  |  |  |
| *PiK3CD* | | *1* | - |  |  |
| *PiK3R1* | | *1* | - |  |  |
| *PiK3CA* | | *1* | - |  |  |
| *MTOR* | | *1* | - |  |  |
|  | |  |  |  |  |
| **ALL Pi3K** | | 8 | - | 15% | (n=7) 19% |
|  | |  |  |  |  |
|  | |  |  |  |  |
| **p53** | |  |  |  |  |
| p53 | | 6 | - | 12% | 16% |
|  | |  |  |  |  |
|  | |  | | | |
| **Others** | |  | | | |
| SPOP | | | ERBB2 | TSHR^#^ | XPO1 |
| PMS2^#^ | | | MED12 | CTNNB | SMAD |
| EPHA5 | | | SDHC^#^ | WT1 | EGFR |
| SMO1 | | | ESR1 | KRAS | p63 (2) |
| SRP1 | | | PPP2R2A | NTRK3 | INSR |
| BCOR | | | CREBBP | SPEN^#^ | TBX3 |
| STK40 | | |  |  |  |
|  | | |  |  |  |

**Supplementary Table 2 –** Pathogenic mutations detected in prostate biopsy samples and actionable status. Percentage of cases with mutations relative to the whole cohort (n=52) and only men with NICE Cambridge Prognostic Group 4-5 or metastatic disease (n=37) are shown. *Actionable based on drug available and trial outcomes already published or in phase 3 clinical trials. # present in germline.
